# Elevated serum HER‐2 predicts poor prognosis in breast cancer and is correlated to ADAM10 expression

**DOI:** 10.1002/cam4.1859

**Published:** 2019-01-19

**Authors:** Hui Zheng, Ailing Zhong, Suhong Xie, Yanchun Wang, Jiajun Sun, Jie Zhang, Ying Tong, Miaomiao Chen, Guihong Zhang, Qian Ma, Jinyan Kai, Lin Guo, Renquan Lu

**Affiliations:** ^1^ Department of Clinical Laboratory Fudan University Shanghai Cancer Center Shanghai China; ^2^ Department of Oncology Shanghai Medical College, Fudan University Shanghai China

**Keywords:** ADAM10, breast cancer, HER‐2, progression‐free survival

## Abstract

Human epidermal growth factor receptor‐2 (HER‐2) overexpression in breast tumor tissues is associated with a poor prognosis but may benefit from treatment with trastuzumab. The extracellular domain (ECD) of HER‐2 can be measured in serum and which has been a new inspection item in clinical laboratory of several hospitals. However, whether serum HER‐2 ECD can be a marker of HER‐2 status in tumor tissues still confused clinicians. This study is a retrospective observation to explore the correlation between serum HER‐2 ECD shedding and tissue HER‐2 status in breast cancer patients. Meanwhile, we will further uncover the potential clinical significance of serum HER‐2 ECD detection. A total of 545 unselected breast cancer patients from Fudan University Shanghai Cancer Center were enrolled in this study. At primary diagnosis without any treatment, serum HER‐2 ECD was measured on ADVIA Centaur assay; meanwhile, tissue HER‐2 from core needle biopsy was tested through immunohistochemistry (IHC) and fluorescent in situ hybridization (FISH). We showed that serum HER‐2 ECD concentration was related to tissue HER‐2 status. Nevertheless, 36.9% of patients with tissue HER‐2 overexpression had low levels of HER‐2 ECD shedding (<15 ng/mL) in serum. Here, we demonstrated that HER‐2 ECD shedding was also associated with protein expression and alpha‐secretase activity of a disintegrin and metalloproteinase 10 (ADAM10) using tumor tissues and cell lines. Progression‐free survival (PFS) data from breast cancer patients in TNM phase II and III with tissue HER‐2 IHC 3+ were analyzed using Kaplan‐Meier plotter. The patients with serum HER‐2 ECD above 15 ng/mL had lower progression‐free survival than those with serum HER‐2 ECD <15 ng/mL. Thus, serum HER‐2 ECD could be a biomarker to identify the subgroup of poorer outcome among HER‐2 overexpression breast cancer patients. Inhibition of ADAM10 activity may have potential therapeutic benefit for this most aggressive tumor subgroup.

## 1 INTRODUCTION

Breast cancer is the most frequent type of cancer and the most common cause of cancer‐related mortality among women worldwide.^1^ The heterogeneity of breast cancer resulted in a molecular classification into four subtypes: Luminal A, Luminal B, human epidermal growth factor receptor‐2 (HER‐2) positive, and basal like.^2^ HER‐2 is overexpressed or amplified in 20%‐30% of primary invasive breast cancers.^3^, ^4^ In breast cancer, HER‐2 overexpression is known to be associated with a poor prognosis because HER‐2 would regulate cell proliferation, adhesion, migration, and differentiation.^5^ Due to several HER‐2‐targeted monoclonal antibodies' emerging, these HER‐2 positive patients can benefit from them.^6^ Trastuzumab is the first approved antibody by US Food and Drug Administration (FDA) as a targeted therapy against HER‐2 in breast cancer patients with HER‐2 positive tumor tissues. The treatment with trastuzumab is expensive and tissue HER‐2 negative patients do not benefit from it, so it is important to determine tumor HER‐2 status.^7^

Currently approved methods for HER‐2 testing include immunohistochemistry (IHC) and fluorescent in situ hybridization (FISH) using tumor tissue. A HER‐2‐positive result requires the demonstration of HER‐2 protein overexpression (3+ staining by IHC) or HER‐2 gene amplification by FISH.^8^, ^9^ The accurate identification of HER‐2 status is critical to the appropriate management of breast cancer patients. False negatives will deny patients with HER‐2‐positive breast tumor a life‐extending therapy, whereas false positives will unnecessarily expose patients to toxic and costly treatment. However, the currently available tissue diagnostic methods for HER‐2 have their own limitations, such as discordance between IHC and FISH diagnosis,^10^ inter‐laboratory variability.^11^ In this instance, serum HER‐2 evaluation was generated as a potentially additional diagnosis for HER‐2 expression.

The full‐length HER‐2 protein is a 185 kDa transmembrane receptor composed of an internal tyrosine kinase domain, a short transmembrane domain, and extracellular domain (ECD).^12^ The HER‐2 ECD has been shown to be shed from cancer cells into the bloodstream where it can be measured in serum.^13^ In theory, serum HER‐2 ECD can be measured serially, and might monitor on‐treatment response, predict relapse, or provide a real‐time assessment of HER‐2 status at metastatic presentation.^14^ However, the clinical utility of serum HER‐2 ECD is very controversial.^15^, ^16^ Whether serum HER‐2 ECD can be a marker of HER‐2 status in tumor tissues and the defined clinical significance confused clinicians. Here, we will explore correlation between serum HER‐2 ECD shedding and HER‐2 expression status in breast tumor tissues. Furthermore, we will uncover the potential clinical significance of serum HER‐2 ECD detection.

## 2 MATERIALS AND METHODS

### 2.1 Study subjects

The patients with malignant or benign breast diseases in the present study were performed an operation in Fudan University Shanghai Cancer Center. Our study is a retrospective study on correlation between tumor tissue HER‐2 and serum HER‐2 ECD at primary diagnosis. Total 545 breast cancer patients (median age, 49 years; range, 26‐83 years) measured for serum HER‐2 ECD and tumor tissue HER‐2 during the period 2015‐2016 were enrolled in. Seventy‐five healthy persons (median age, 39 years; range, 28‐60 years) and 118 patients with benign breast diseases (median age, 47 years; range, 31‐72 years) were used as control groups. All participants were informed and gave their written consent. The study's protocol was reviewed and approved by the Ethics Committee of Fudan University Shanghai Cancer Center (certification No. 050432‐4‐1212B).

### 2.2 Tissue HER‐2 testing

Tissue HER‐2 from core needle biopsy was tested near the period of blood collecting. Determining of tissue HER‐2 expression was performed by clinical pathologists depending on IHC and FISH. IHC 3+ or IHC 2+ and FISH + were defined as tissue HER‐2 positive.

### 2.3 Cell culture and ADAM10 inhibitor treatment

Human breast cancer cell lines SK‐BR‐3 (HER‐2 overexpression subtype) and BT‐474 (Luminal B subtype) were obtained from Shanghai Institute of breast cancer. The cells were maintained in RPIM 1640 medium (Gibco, Waltham, MA, USA) supplemented with 10% Fetal calf serum (Gibco, Brazil) and Penicillin‐Streptomycin (Solarbio, Shanghai, China). Protein of a disintegrin and metalloproteinase 10 (ADAM10) inhibitor, GI254023X, was purchased from Sigma (St. Louis, MO, USA, product#: SML0789) and resuspended in DMSO (Sigma) to 20 mmol/L stock. Cells were then treated with ADAM10 inhibitor or DMSO vehicle to detect the influence on HER‐2 ECD shedding.

### 2.4 Measurement of HER‐2 ECD

The concentration of HER‐2 ECD was measured on ADVIA Centaur CP Immunoassay System (Siemens, Germany). For serum HER‐2 ECD analysis, the cutoff value is 15 ng/mL according to manufacturer's manual and which is used as reference toplimit in clinical application. Serum HER‐2 ECD ≥15 ng/mL was regarded as positive. Cell HER‐2 ECD was measured in cell culture supernatant.

### 2.5 ADAM10 staining and IHC scoring

ADAM10 expression levels were detected using IHC on breast tumor tissues with HER‐2 IHC 3+. The staining procedure was described previously.^17^ Rabbit polyclonal antibody to ADAM10 was purchased from Abcam (Boston, MA, USA, ab1997, 1:500) using as primary antibody. The scoring criteria are a composite score based on staining intensity and percentage of positive cells using the formula proposed by Ruiter et al^18^ The percentage of positive tumor cells was scored as 0 for <1%; 1 for 1%‐5%; 2 for 6%‐25%; 3 for 26%‐50%; 4 for 51%‐75%; and 5 for >75%. The intensity of staining was scored using the following scale: 0, negative; 1, weak; 2, moderate; 3, strong. A final score was calculated by totaling the scores for percentage and intensity. A final score of 0 indicated negative expression, 2‐4 weak expression, 5‐6 moderate expression, and 7‐8 strong expression.

### 2.6 Cell alpha‐secretase activity detection

The alpha‐secretase activity of breast cancer cells with or without ADAM10i treatment was detected by a fluorescence resonance energy transfer kit (GENMED, Shanghai, China), according to the manufacturer's instructions. In brief, 100 μg protein extraction from cells was mixed with substrate in 96‐well black plate, then relative fluorescence unit was detected on BioTek SynergyH4.

### 2.7 Western blot

The cells in culture plates were collected and lysed, then the proteins were separated on a 8% acrylamide gel by SDS‐PAGE. When the separated proteins were transferred to polyvinylidene fluoride membranes and blocked with 5% milk in Tris‐buffered saline, anti‐ADAM10 (ab1997, 1:1000) or anti‐ErbB 2 antibody (CB11, 1:1000) from Abcam (Boston, MA, USA) was incubated respectively at 4°C overnight. The membranes were washed with Tris‐buffered saline and Tween 20 solution. HRP‐conjugated goat anti‐rabbit (1:5000 for ADAM10) or goat anti‐mouse (1:5000 for ErbB 2) secondary antibody was purchased from SAB (Signalway Antibody, MA, USA). The blots were visualized with a chemiluminescent substrate (Thermo Scientific, Rockford, IL, USA) and exposed to Amersham Imager 600 (GE Healthcare, PA, USA).

### 2.8 Cell surface HER‐2 assay

Surface HER‐2 expression in cultured cells was detected by flow cytometry with anti‐human p185 HER‐2/FITC (Invitrogen, Vienna, Austria). The data were acquired on a FACSCalibur (BD Biosciences, NJ, USA), and analyzed using FlowJo software (Ashland, OR, USA).

### 2.9 Statistical analysis

The positive proportion of serum HER‐2 ECD expression in breast cancer patients was compared with which in healthy and benign controls using chi‐square analysis. Mann‐Whitney *U* test was applied to analysis the serum HER‐2 ECD levels between two groups. The differences of cell HER‐2 ECD between two means were assessed by an independent Students’ *t* test (two‐tailed). Progression‐free survival (PFS) data were analyzed using Kaplan‐Meier plotter. Statistical analysis was carried out with SPSS version 16.0 software (IBM Corporation, NY, USA). Differences were considered significant when *P* value was <0.05.

## 3 RESULTS

### 3.1 The correlation between serum HER‐2 ECD expression and tumor tissue HER‐2 status at primary diagnosis

**Figure 1:**
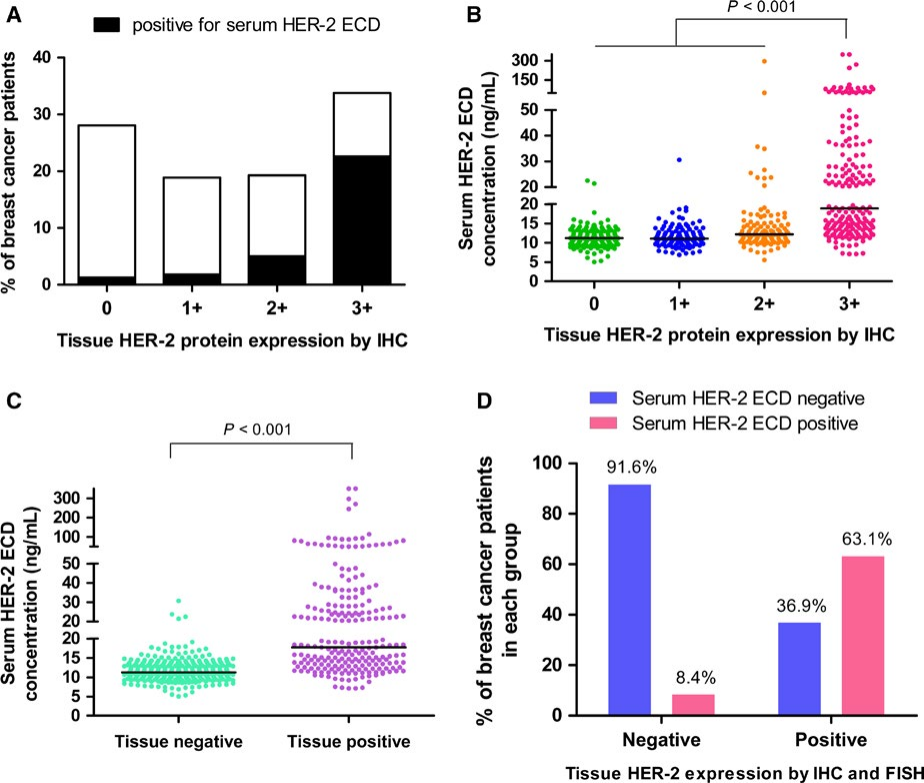
Serum HER‐2 ECD levels and tumor tissues HER‐2 status in 545 primary breast cancer patients. A, The positive rate of serum HER‐2 ECD expression in breast cancer patients with different degrees of IHC staining. B, Serum HER‐2 ECD concentration of patients with different degrees of IHC staining. C, Serum HER‐2 ECD expression levels in tissue HER‐2 negative and positive patients. D, The expression concordance between serum HER‐2 ECD and tissue HER‐2 status

HER‐2 ECD generated as a potentially additional diagnosis for HER‐2 expression, whether it can be used to determine HER‐2 expression status? Thus, serum HER‐2 ECD levels from 545 primary breast cancer at diagnosis without any treatment were analyzed. In Table 1, 30.6% (167/545) of breast cancer patients showed positive serum HER‐2 ECD expression (≥15 ng/mL), while 5% (6/118) of benign and 4% (3/75) of healthy controls were positive, respectively. These data demonstrated that the specificity of serum HER‐2 ECD for diagnosis of breast cancer was high. Meanwhile, tissue HER‐2 status in 545 breast cancer patients was compared with serum HER‐2 ECD levels. IHC is a most common method for HER‐2 protein testing, and the serum HER‐2 ECD‐positive rate was correlated positively with IHC staining intensity (Figure 1A). Serum HER‐2 ECD levels in patients with HER‐2 IHC 3+ were significantly higher than others with different degrees of IHC staining (Figure 1B). If the tissues with IHC 3+ or IHC 2+ and FISH + were defined as tissue HER‐2 positive, our data indicated that serum HER‐2 ECD concentration in tissue HER‐2‐positive patients was increased significantly as compared with tissue HER‐2‐negative patients (Figure 1C). The concordance between serum HER‐2 ECD and tissue HER‐2 status was also analyzed. The percentage of serum HER‐2 ECD‐negative in tissue HER‐2‐negative patients (negative concordance rate) is high (91.6%); however, the positive concordance rate is low (63.1%; Figure 1D).

**Table 1:** Serum HER‐2 ECD expression in healthy persons and patients with malignant or benign breast diseases

	Serum HER‐2 ECD concentration		*P*‐value
	<15 ng/mL	≥15 ng/mL	
Malignant (n = 545)	378 (69.4%)	167 (30.6%)	<0.001[Link]
Benign (n = 118)	112 (95%)	6 (5%)	
Healthy (n = 75)	72 (96%)	3 (4%)	

The positive proportion of serum HER‐2 ECD expression in breast cancer patients was significantly higher compared with which in healthy and benign controls.

### 3.2 Serum HER‐2 ECD shedding is correlated to ADAM10 expression

**Figure 2:**
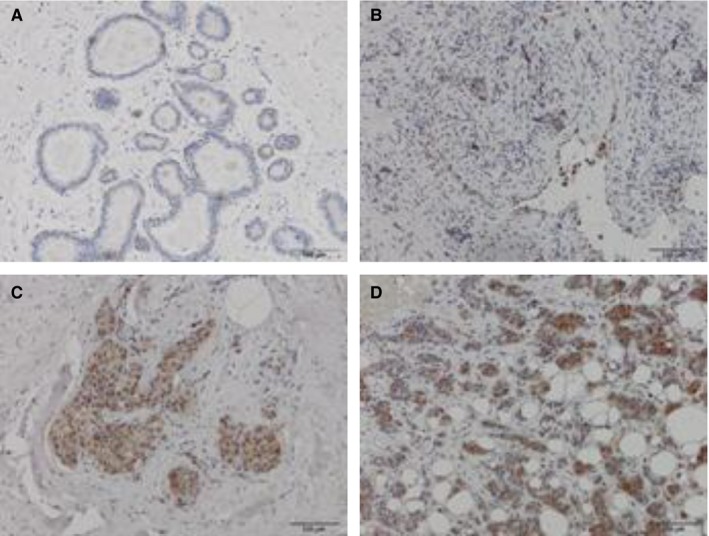
Samples with different staining intensities against ADAM10 protein in breast tumor tissues with HER‐2 IHC 3+. A and B, Negative and weak ADAM10 expression in patients with negative‐sHER‐2 ECD. C and D, Moderate and strong ADAM10 expression in patients with positive‐sHER‐2 ECD. Bar (right bottom) represents 100 μmol/L

To further clarify that the low serum HER‐2 ECD‐positive rate in tissue HER‐2‐positive patients is caused by low sensitivity of serum HER‐2 ECD assay system or just a physiological situation, we set out to research unidentified ectodomain sheddase. Some studies in HER‐2 shedding cell lines have implicated ADAM10 being a major source of HER‐2 sheddase activity.^19^ Therefore, we tested tissue ADAM10 expression in breast cancer patients with HER‐2 IHC 3+. Table 2 summarizes the ADAM10 protein expression in both groups. Nearly, all of the samples in patients with serum HER‐2 ECD‐negative (18/20) showed negative or weak ADAM10 expression, while 80% of samples in patients with serum HER‐2 ECD‐positive (16/20) showed moderate or strong ADAM10 expression. Examples of negative and positive ADAM10 expression patterns are depicted in Figure 2.

**Table 2:** ADAM10 expression in breast cancer patients with different HER‐2 expression pattern

ADAM10 expression in breast cancer tissues	HER‐2 positive expression in breast cancer tissues	
	Serum HER‐2 ECD‐negative	Serum HER‐2 ECD‐positive
Negative	2	0
Weak	16	4
Moderate	2	12
Strong	0	4

### 3.3 Specific alpha‐secretase activity inhibitor of ADAM10 reduced HER‐2 shedding

**Figure 3:**
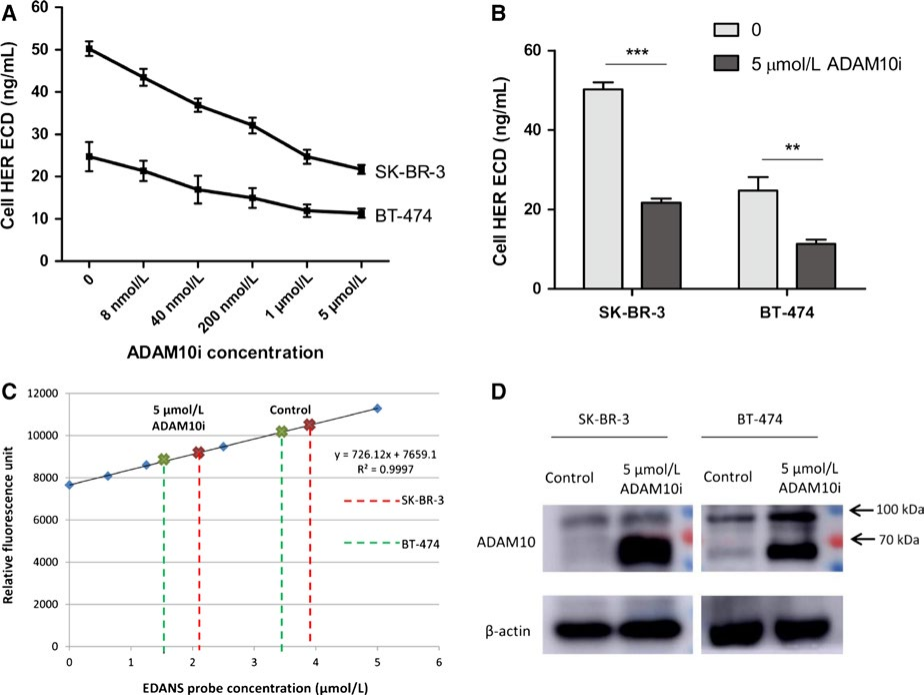
ADAM10i could reduce HER‐2 ECD levels in medium supernatant of HER‐2‐positive breast tumor cells. A, Cells (2.5 × 10^5^) were cultured in 24‐well plate (500μL medium/well) with different levels of ADAM10i for 48 hours, then the medium supernatant was collected for HER‐2 ECD detection. B, HER‐2 ECD concentration with 5 μmol/L ADAM10i was reduced significantly. **P < 0.01, ***P < 0.001. C, To verify the inhibition effect of ADAM10i on a‐secretase. D, ADAM10i upregulated total ADAM10 protein levels because of a negative feedback. Control, cells treated with DMSO vehicle. 5 μmol/L ADAM10i, culture for 48 hours

**Figure 4:**
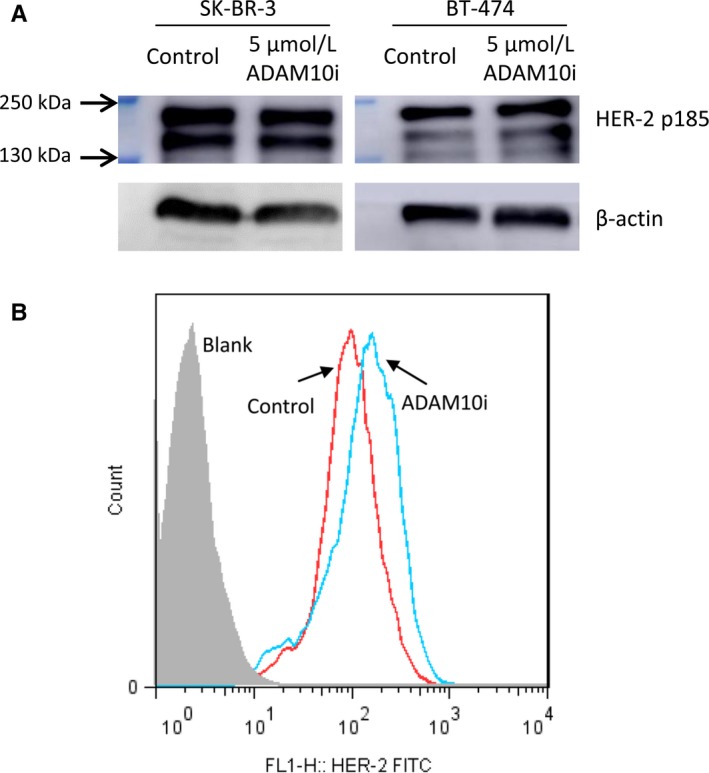
The effect of ADAM10i on full‐length HER‐2 protein and cell surface HER‐2 expression. A, There was no influences to full‐length HER‐2 protein levels with ADAM10i. B, ADAM10i up‐regulated HER‐2 expression levels on cell surface. Control, cells treated with DMSO vehicle. 5 μmol/L ADAM10i, culture for 48 hours

In order to directly demonstrate HER‐2 ECD shedding is regulated by ADAM10, HER‐2 shedding cell lines SK‐BR‐3 and BT‐474 were used. Liu et al have verified in SK‐BR‐3 cells that transfection of ADAM10 siRNA leading to reduction in HER‐2 ECD. Here, we wonder the exact function of protein ADAM10 associated with HER‐2 ECD shedding. GI254023X (ADAM10i) is a chemical inhibitor to specific inhibit alpha‐secretase of ADAM10. As shown in Figure 3A, ADAM10i could downregulate HER‐2 ECD in cell medium depending on ADAM10i concentration, and the maximum inhibition rate was achieved at 5 μmol/L ADAM10i (Figure 3B). Interestingly, ADAM10i could downregulate alpha‐secretase activity of both cells (Figure 3C), while the total ADAM10 protein expression was upregulated (Figure 3D). In our opinions, elevated ADAM10 protein expression may be a negative feedback to inhibition of ADAM10 function, and further indicated that HER‐2 ECD shedding was exactly associated with alpha‐secretase activity of ADAM10. Meanwhile, full length of HER‐2 (p185) showed no difference thereby excluding the possibility that ADAM10i reduced HER‐2 protein levels nonspecifically (Figure 4A). However, HER‐2 expression levels on cell surface were increased a bit using ADAM10i (Figure 4B), which was contrast to reduced HER‐2 ECD with ADAM10i in cell medium. Moreover, the cell counts at the time of HER‐2 ECD detection did not show reduced cell numbers with ADAM10i.

### 3.4 High expression of HER‐2 ECD is associated with decreased progression‐free survival

**Figure 5:**
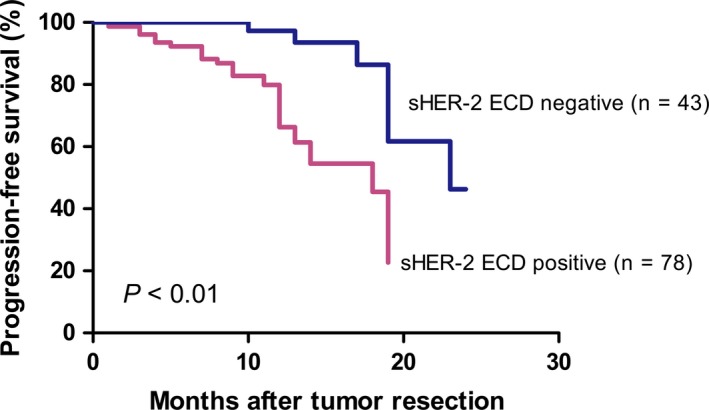
Progression‐free survival (PFS) data from breast cancer patients in TNM phase II‐III with tissue HER‐2 IHC 3+. Serum HER‐2 ECD‐positive patients had a poorer outcome compared with serum HER‐2 ECD‐negative cohort

Since that the low positive concordance rate between serum HER‐2 ECD and tissue HER‐2 status at primary diagnosis is just a physiological situation, how about the clinical value of serum HER‐2 ECD detection? We analysis PFS data from breast cancer patients in TNM phase II and III with tissue HER‐2 IHC 3+ and having a similar treatment options including neoadjuvant chemotherapy, operation and trastuzumab therapy. Any pathologic changes such as tumor biomarker increasing, pulmonary or liver nodule, bone or brain metastasis were all regarded as progression. As shown in Figure 5, the cohort of patients with serum HER‐2 ECD‐positive had a poorer outcome compared with serum HER‐2 ECD‐negative patients.

## 4 DISCUSSION

HER‐2 ECD is a prognosis factor that is valuable in evaluating the neoadjuvant treatment efficiency.^20^ HER‐2 ECD also appears to be a helpful surveillance biomarker for the early diagnosis of relapses and to predict the fate of metastases.^15^ Our present paper indicated that HER‐2 ECD had a high specificity to distinguish breast cancer patients from benign (5% increased HER‐2 ECD) and healthy controls (4% increased HER‐2 ECD). The positive proportion of HER‐2 ECD among unselected women with breast cancer in our study cohort is 30.6%, corresponding to reported percentage of HER‐2 overexpression in breast tumor tissues. Thus, HER‐2 ECD may also be a breast cancer biomarker. However, serum HER‐2 ECD and tissue HER‐2 expression are not completely matched. Especially, 36.9% of patients with tissue HER‐2 overexpression showed normal HER‐2 ECD value (<15 ng/mL). The sensitivity of serum HER‐2 ECD for diagnosis of HER‐2‐positive breast cancer is somewhat poor. Therefore, we confirm some authors' proposition that serum is not a good alternative to histological determination of tissue HER‐2 status.^21^

Protein shedding via various ADAMs is important for cell fate determination, migration, and proliferation.^22^ Liu et al^19^ showed that ADAM10 was a major source of HER‐2 ectodomain sheddase activity in HER‐2 overexpressing breast cancer cells. In this regard, ADAM10 expression differences were verified between serum HER‐2 ECD‐positive and ‐negative patients with tissue HER‐2 IHC 3+. The negative or weak expression of ADAM10 could explain for the low serum HER‐2 ECD levels in patients with tissue HER‐2 overexpression. We also identified the relationship between alpha‐secretase activity of ADAM10 and HER‐2 ECD shedding in HER‐2‐positive cell lines. Inhibition of ADAM10 activity could reduce HER‐2 ECD shedding.

HER‐2 overexpression or amplification has been associated with a more aggressive disease, a poor clinical prognosis.^23^, ^24^ Our study showed that elevated serum HER‐2 ECD could be associated to a more aggressive clinical course. There are several explanations for the link between HER‐2 ECD level and PFS. Firstly, the binding of therapeutic anti‐HER‐2 antibodies on HER‐2 ECD neutralizes the biological activity of trastuzumab.^25^ Furthermore, amounts of HER‐2 ECD shedding leaves a truncated HER‐2 form (p95) with constitutive kinase activity that can provide ligand‐independent growth and survival signals to the cells.^26^ Therefore, serum HER‐2 ECD could be a biomarker that helps to identify the subgroup of poorer outcome among HER‐2 overexpression breast cancer patients.

In summary, we revealed that serum HER‐2 ECD was not completely matched with tissue HER‐2 status because of the differential expression of ADAM10. Overexpression of ADAM10 resulting in increased HER‐2 ECD shedding is associated with poorer prognosis. Inhibition of ADAM10 activity may have potential therapeutic benefit for this most aggressive tumor subgroup.

## CONFLICT OF INTEREST

The authors declare that they have no conflict of interest.
